# Phage-Host Prediction Using a Computational Tool Coupled with 16S rRNA Gene Amplicon Sequencing

**DOI:** 10.3390/v15010076

**Published:** 2022-12-27

**Authors:** Harilanto Felana Andrianjakarivony, Yvan Bettarel, Fabrice Armougom, Christelle Desnues

**Affiliations:** 1 Microbes, Evolution, Phylogeny, and Infection (MEΦI), IHU—Méditerranée Infection, 19-21 Boulevard Jean Moulin, 13005 Marseille, France; 2 Microbiologie Environnementale Biotechnologie (MEB), Mediterranean Institute of Oceanography (MIO), 163 Avenue de Luminy, 13009 Marseille, France; 3 MARBEC, Marine Biodiversity, Exploitation & Conservation, Université de Montpellier, CNRS, Ifremer, IRD, 093 Place Eugène Bataillon, 34090 Montpellier, France

**Keywords:** virome, phage-host interaction, 16S rRNA metabarcoding, host prediction, bacteria, metagenomic, lagoon

## Abstract

Metagenomics studies have revealed tremendous viral diversity in aquatic environments. Yet, while the genomic data they have provided is extensive, it is unannotated. For example, most phage sequences lack accurate information about their bacterial host, which prevents reliable phage identification and the investigation of phage–host interactions. This study aimed to take this knowledge further, using a viral metagenomic framework to decipher the composition and diversity of phage communities and to predict their bacterial hosts. To this end, we used water and sediment samples collected from seven sites with varying contamination levels in the Ebrié Lagoon in Abidjan, Ivory Coast. The bacterial communities were characterized using the 16S rRNA metabarcoding approach, and a framework was developed to investigate the virome datasets that: (1) identified phage contigs with VirSorter and VIBRANT; (2) classified these contigs with MetaPhinder using the phage database (taxonomic annotation); and (3) predicted the phages’ bacterial hosts with a machine learning-based tool: the Prokaryotic Virus-Host Predictor. The findings showed that the taxonomic profiles of phages and bacteria were specific to sediment or water samples. Phage sequences assigned to the *Microviridae* family were widespread in sediment samples, whereas phage sequences assigned to the *Siphoviridae*, *Myoviridae* and *Podoviridae* families were predominant in water samples. In terms of bacterial communities, the phyla *Latescibacteria*, *Zixibacteria*, *Bacteroidetes*, *Acidobacteria*, *Calditrichaeota*, *Gemmatimonadetes*, *Cyanobacteria* and *Patescibacteria* were most widespread in sediment samples, while the phyla *Epsilonbacteraeota*, *Tenericutes*, *Margulisbacteria*, *Proteobacteria*, *Actinobacteria*, *Planctomycetes* and *Marinimicrobia* were most prevalent in water samples. Significantly, the relative abundance of bacterial communities (at major phylum level) estimated by 16S rRNA metabarcoding and phage-host prediction were significantly similar. These results demonstrate the reliability of this novel approach for predicting the bacterial hosts of phages from shotgun metagenomic sequencing data.

## 1. Introduction

Bacteriophages (viruses that infect bacteria) make up the majority of viruses found on Earth and occur in a variety of environments: marine, freshwater and terrestrial [1,2]. Phages are likely to be distributed wherever their potential hosts exist [3]. They have been found to have a significant impact on microbial ecosystems by affecting bacterial mortality, reshaping bacterial diversity via horizontal gene transfer and rewiring bacterial metabolism [4]. Phages are classified as lytic (virulent) or lysogenic (temperate) depending on the infection pathway they use when targeting a permissive host [5]. As our knowledge of viral diversity increases, new tools are needed to facilitate the identification of newly discovered viruses, allowing taxonomic and functional assignment and the prediction of their associated hosts. The identification of the viral host is essential for the characterization of phages, as they depend on the host for survival [3]. Currently, the most common method used to determine the host of a phage is through cultures, but this can be inefficient, time consuming and expensive [6].

In recent decades, shotgun metagenomic sequencing has been proposed to study genomes of uncultured viral populations in the environment, an approach known as viral metagenomics [7]. This has allowed virome analysis (of all viral assemblies in a given environment) to uncover many new phage genomes never previously reported, enriching viral sequence databases [7]. However, in contrast to the conventional culture-based approach, which provides direct host information, viral metagenomics does not reveal the relationships between phages and their hosts [8]. This has led to a growing demand for computational tools able to annotate new viral genomes with host taxon information [9]. To date, high-throughput methods for determining reliable virus-host associations are lacking, preventing this aspect from keeping pace with the rapid pace of virus discovery.

Several approaches have been put forward to predict phage-host relationships; for the most part, these are based on abundance profiles, genetic homology, CRISPR, exact matches or oligonucleotide profiles [8,10]. More recently, a variety of computational approaches have been developed for phage-host prediction. These fall under two main groups depending on their use of sequence alignment: alignment-based or alignment-free methods [11]. The alignment-based methods (e.g., using BLAST or CRISPR spacers) rely on sequence-similarity searches between a query virus and a host genome, since viruses and hosts may share genes and short nucleotide sequences [8,12]. Alignment-free methods predict the host of a virus based on the co-occurring k-mers (the oligomers of length *k*) of phages with known hosts [13] or the similarity of sequence signatures between viruses and their hosts [8]. To determine the most likely host, these methods calculate the similarity between the phage sequence and the genome of each candidate host using oligonucleotide frequency, a Markov chain model, or a Gaussian model [10]. Of the latter approaches, VirHostMatcher [12] and WIsH [14] have shown the highest accuracy in predicting hosts. 

For this study, we chose the Prokaryotic Host Predictor (PHP) (https://github.com/congyulu-bioinfo/PHP) as it has greater prediction accuracy than VirHostMatcher and WIsH (28–34%, genus level) [11]. This software tool uses a Gaussian model to predict the hosts of prokaryotic viruses by looking for differences in k-mer frequencies between viral and host genomic sequences [11]. K-mer profiles use nucleotide composition to predict the host of a viral sequence by identifying the corresponding prokaryotic genome with the highest significant similarity, assuming that this is the host of the virus of interest [15]. 

In a previous study, we had characterized the viral communities of the Ebrié Lagoon in Abidjan in the Ivory Coast, a tropical lagoon subject to high levels of contamination from human activities (submitted for publication 2022). Most of the sequences obtained from the virome analysis were annotated as phages. However, because studies of viruses in tropical lagoon environments are scarce, phage hosts remain largely unknown. To investigate this further, we aimed to develop a viral metagenomic framework able to describe the phage communities and predict their putative bacterial hosts. The viromic data was obtained from water and sediment collected from seven sites with contrasting contamination levels from the lagoon. In parallel to the metagenomic framework, we performed a 16S metabarcoding analysis to describe the bacterial communities. This allowed us to compare the relative abundance of bacterial communities (at phylum level) estimated by the 16S-based method and by phage-host prediction to assess the accuracy of the viral metagenomic framework in predicting phage hosts and to identify its main challenges.

## 2. Materials and Methods

### 2.1. Study Site and Sample Collection

The samples were collected between 21 and 28 May 2019 around the Ebrié Lagoon, which borders Abidjan, the Ivory Coast’s largest city. Seven stations (S) with contrasting anthropization levels and sources were sampled around the lagoon: (S1) Mondoukou (05°11′15.8″ N, 03°41′20.8″ W), (S2) Cocody Bay (05°19′41.1″ N, 03°59′26.4″ W), (S3) Yopougon Beach (05°18′26.0″ N, 04°02′18.0″ W), (S4) Yopougon Bay (05°18′31.1″ N, 04°04′08.3″ W), (S5) Boulay Island (05°17′15.0″ N, 04°01′48.6″ W), (S6) Bietri slaughterhouse (05°15′58.3″ N, 03°58′01.1″ W) and (S7) Bietri Neck (05°15′37.0″ N, 03°58′28.8″ W). Sediment and water samples were collected in triplicate at each of the seven stations. Water samples were collected at a depth between 15 and 50 cm using 1.5-L sterile plastic Nalgene bottles. The bottles were rinsed twice with lagoon water before collection. Sediment samples were collected with a Van Veen grab (KC Denmark) at a depth of approximately 1 m. The top layer of each sediment core (~2 cm) was removed and placed in a sterile plastic ziplock bag (Whirl-Pak). All samples were kept cool in an icebox during transport to the laboratory, where they were analyzed within 3 h of collection.

### 2.2. Bacterial DNA Extraction, Amplification and Sequencing

The three water and sediment samples taken from each station were analysed separately (n_wat_ = 21; n_sed_ = 21). Water samples (100 mL) were filtered through 0.2 µm filters (Anodisc, Whatman, Maidstone, UK). The filters and sediment samples were transferred into cryotubes, flash-frozen in liquid nitrogen, and stored at −80 °C prior to nucleic acid extraction. DNA extraction was performed using the PowerSoil^®^DNA Isolation Kit (MoBio Laboratories, Solana Beach, CA, USA) following the manufacturer’s instructions. 

The DNA was quantified by fluorescence using the Qubit dsDNA BR Assay kit (Invitrogen, Carlsbad, CA, USA) and the Qubit 3.0 Fluorometer. The universal primer set 341F (5′-CCTACGGGNGGCWGCAG-3′) and 785R (5′-GACTACHVGGGTATCTAATCC-3′) was used to amplify a 444-bp fragment size corresponding to the V3–V4 region of the 16S rRNA gene [16]. The reaction was carried out in a 25-µL mixture including 0.5 μL of each primer at 10 μM, 12.5 μL of 2X KAPA HiFi HotStart ReadyMix (KAPA Biosystems Inc., Wilmington, MA, USA), 2.5 µL of DNA template (0.5 µg/mL), and 9 µL of sterilized water. The following PCR conditions were applied: initial denaturation at 94 °C for 3 min, followed by 25 cycles of 95 °C for 30 s, 55 °C for 30 s, and 72 °C for 30 s, ending with a final extension at 72 °C for 5 min. The PCR products were purified with Agencourt AMPure beads (Beckman-Coulter, Villepinte, France) following the manufacturer’s protocol. The quality of the PCR products was checked by agarose gel electrophoresis. The resulting amplicons were quantified by fluorescence using the Qubit dsDNA BR Assay kit (Invitrogen, Carlsbad, CA, USA) and the Qubit 3.0 Fluorometer. The DNA quality was checked with the Agilent DNA 7500 kit on the Agilent 2100 Bioanalyzer System (Agilent Technologies, Santa Clara, CA, USA) following the manufacturer’s protocol. The amplicons were sequenced with MiSeq Technology using the Nextera XT library kit in a 2 × 250 bp format (Illumina Inc., San Diego, CA, USA).

### 2.3. 16S rRNA Gene Sequence Analysis

Bioinformatic analyses were performed using RStudio (v2021.9.0) and R version 4.1.2 (Figure S1). Raw reads were preprocessed using DADA2 v1.22.0, a model-based approach for correcting sequencing errors [17]. The 16 rRNA paired-end reads were quality checked, trimmed, dereplicated, denoised, assembled and the chimeras were discarded following the DADA2 pipeline [17]. The high-quality sequences obtained were considered amplicon sequence variants (ASVs), in which each ASV differs from the others by at least one nucleotide. The taxonomic assignment of ASVs was performed using the SILVA database, version 132 [18], with 100% of sequence identity required for species ranking. The final ASV abundance table was normalized by subsampling for downstream analysis. Beta diversity was characterized using the R packages phyloseq v1.32 [19], vegan v2.5, and pheatmap v1.012. The visualization and comparison of the taxonomic profiles of the bacterial communities in the water and sediment samples from the seven stations were performed by hierarchical clustering using the Bray-Curtis dissimilarity [20].

### 2.4. Viral Particles Isolation and Viral Metagenomic Analysis

The viral DNA extraction and viral metagenomic analyses have been detailed in a previous article (submitted for publication 2022). The viral particles were isolated and purified from the water and sediment samples of the seven stations by particle-size filtration and sucrose centrifugation. The total viral nucleic acids were extracted and purified using the Roche High Pure Viral Nucleic Acid Kit (Roche Diagnostics, Basel, Switzerland) following the manufacturer’s protocol. The DNA was amplified in duplicate using a Genomiphi Kit (GE Healthcare, Chicago, IL, USA) following the manufacturer’s protocol. The resulting DNA was sequenced using MiSeq technology (next-generation sequencing). Viral metagenomic framework is represented in Figure S1. The quality control of reads was carried out using Trimmomatic [21], AfterQC [22] and FASTQc [23]. The resulting reads were assembled with MetaSpades [24]. Contig assemblies were aligned with DIAMOND [25] using BLASTx against the non-redundant (nr) NCBI GenBank protein database with an e-value of 10^−3^. Taxonomic annotation was performed with MEGAN-CE (MEtaGenome Analyzer; v.6.3) [26] using the lowest common ancestor (LCA) algorithm with a min-score of 50, a top-percent filter of 0.001 and a min-support filter of 1. Contigs annotated as phages were exported and processed for taxa relative abundance analysis. The visualization and comparison of the taxonomic profiles of the phage communities in the water and sediment samples from the seven stations were performed by hierarchical clustering using the Bray-Curtis dissimilarity [20].

### 2.5. Identification and Classification of Phage Contigs

To predict phage contigs from virome datasets, we used the CyVerse Discovery Environment platform (https://de.cyverse.org, accessed on 7 January 2020) to run VirSorter v1.0.3 [27] and VIBRANT v1.2.0 [28]. The used contigs represent genome fragments with a minimum length of 1000 base pairs (bp) and a maximum length of 58,536 bp. The minimum contig length was chosen to obtain reliable phage contigs (partial genomes) for estimating the associated host, as viral genomes vary considerably in length. No maximum size requirement was imposed to increase the possibility of having complete phage genomes. VirSorter annotates contigs using MetaGeneAnnotator [29], and then uses hmmsearch [30] to predict PFAM domains [31] and viral domains on the annotated genes. VirSorter was run in decontamination mode with the virome database. Predicted phages were assigned to categories (from 1 to 6). We retained phages in categories 1 to 3, which corresponded to the “most reliable” (1), “likely” (2), and “possible” (3) predictions. As a second complementary approach, we used VIBRANT, a hybrid machine-learning and protein-similarity tool that allows the automated recovery of both free and integrated phage genomes from metagenome assemblies. Only phage sequences predicted and classified (category 1, 2 and 3) by the two aforementioned tools (VirSorter and VIBRANT) were used for host prediction and were examined with MetaPhinder v.2.1 [32], which compares contigs to a database of the whole genome sequences of phages.

### 2.6. Prediction of Phage Hosts

Putative bacterial hosts were predicted with the Prokaryotic Virus-Host Predictor (PHP) [11], a computational tool for host prediction of prokaryotic viruses based on a Gaussian model (GM). GM for predicting hosts of prokaryotic viruses takes the differences of k-mer frequencies between viral and host genomic sequences as features, and outputs a score (the logarithm of the probability of being viral host) for bacteria [11]. The k-mer frequencies correspond to the number of subsequences (consisting of nucleotides) of length k (k = 4) composing the bacterial and viral genomes. This tool takes the predicted phage contigs as inputs. For each phage contig, PHP calculates the host probability for 60,105 prokaryotic genomes, assigning the prokaryotic genome with the highest probability as the predicted host. The outputs of this tool include the name of the bacterial genome with the highest score as well as the host score of all bacterial genomes. Host prediction was performed at phylum level. The list of bacterial hosts was extracted, and their relative abundance was calculated using an internal Python script. The relative abundance of each bacterial community obtained by PHP was compared to that of the bacterial community obtained by the 16S method. 

### 2.7. Statistical Analysis

Statistical analyses of viromes, 16S rRNA gene sequence, and phage-host predictions were performed using the R packages FSA v0.9.3 and Stat v4.1.2. The significance threshold for the p-value was 0.05. The adjusted p-value for correcting multiple tests was based on the Benjamini-Hochberg procedure [33]. All plots were created using the R package ggplot2 v.3.3.5. To test for significant differences between the taxonomic profiles of bacterial communities in sediment and water samples, permutational multivariate analysis of variance (PerMANOVA) was performed based on the Bray-Curtis distance using the adonis function in the R package vegan. To test the homogeneity of multivariate dispersions (i.e., deviations from centroids) between sample types, a permutation multivariate analysis of dispersion (PERMDISP) was performed using the betadisper and permutest function from the R package vegan. To compare the similarity between the relative abundance of bacterial communities obtained from the two approaches (16S vs phage-host prediction), a correlation test (Spearman’s rank-order correlation) was applied. Spearman R correlation falls in the range of −1 to +1: −1 indicates a perfect negative association of ranks and +1 indicates a perfect positive association. An r value of 0 indicates no association between ranks.

## 3. Results

### 3.1. Taxonomic Profile of Bacterial Communities at Phyla Level in Water and Sediment Samples

**Figure 1:**
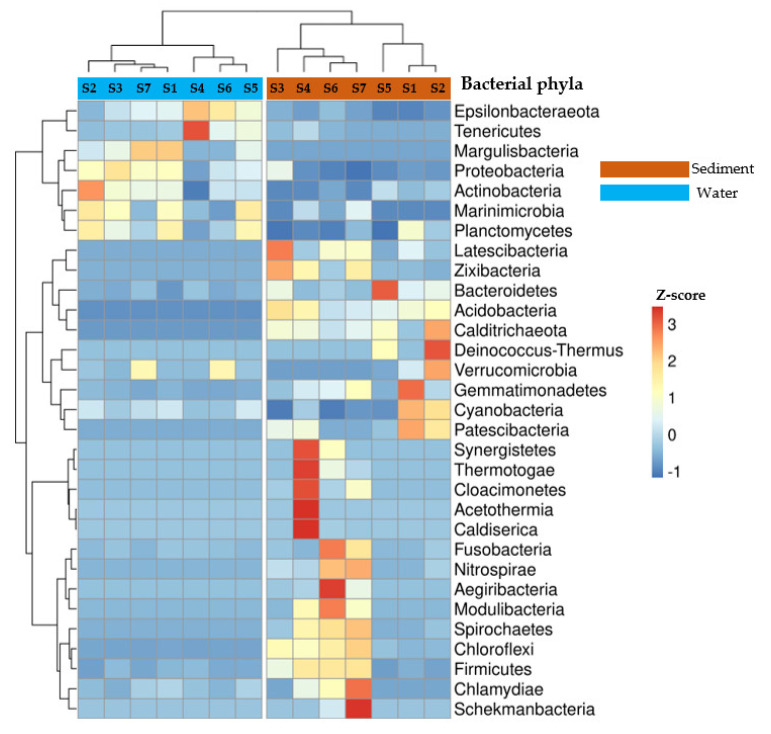
Hierarchical heatmap representing the relative abundance of bacterial communities (at phyla level) in the sediment and water samples of the seven stations (S1–S7). The relative abundance is represented by Z-score (based on the mean and standard-deviation (SD) of Z-scores of each phyla in all samples). The stations and bacterial phyla were clustered using the bray-curtis distance, which is represented by a dendrogram on the top and left side of the graph. Z-scores are scaled according to the relative abundance of bacterial communities. A positive z-score indicates that the value is above average. A z-score of 0 indicates that the value is within the average. A negative z-score indicates that the value is below average.

The hierarchical heatmap showed a distinctive taxonomic profile between bacterial communities in sediment and water samples (Figure 1). The phyla *Latescibacteria*, *Zixibacteria*, *Bacteroidetes*, *Acidobacteria*, *Calditrichaeota*, *Gemmatimonadetes*, *Cyanobacteria* and *Patescibacteria* were most widespread in sediment samples, while the phyla *Epsilonbacteraeota*, *Tenericutes*, *Margulisbacteria*, *Proteobacteria*, *Actinobacteria*, *Planctomycetes* and *Marinimicrobia* were most prevalent in water samples. The sediment in three stations (S4, S6, S7) had a distinct pattern related to the phyla *Chlamydiae*, *Aegiribacteria*, *Fusobacteria*, *Nitrospirae*, *Chloroflexi*, *Firmicutes*, *Modulibacteria* and *Spirochaetes*. Based on a comparison of the Bray-Curtis dissimilarity, the taxonomic profiles of bacterial communities in sediment and water samples were tested by PerMANOVA and were found to be significantly different (F = 8.72, *p* = 0.003) (Figure 1). Dispersion analysis showed that the bacterial communities in water samples were more homogeneous (the communities were more similar between stations) compared to those in sediment samples (the communities were more dissimilar between stations) (Figure S2).

### 3.2. Taxonomic Profile of Phage Communities in Water and Sediment Samples

In terms of phage predictions, VirSorter provided 3631 (sediment) and 9682 (water) putative phage sequences, while VIBRANT recovered 6522 (sediment) and 14,505 (water) phage sequences (Table S2). The taxonomic annotation of the identified phage sequences by METAPHINDER showed a predominance of phages belonging to the *Caudovirales* order (*Siphoviridae*, *Myoviridae* and *Podoviridae* families) in water samples and belonging to the *Microviridae* family in sediment samples (Figure S3). 

**Figure 2:**
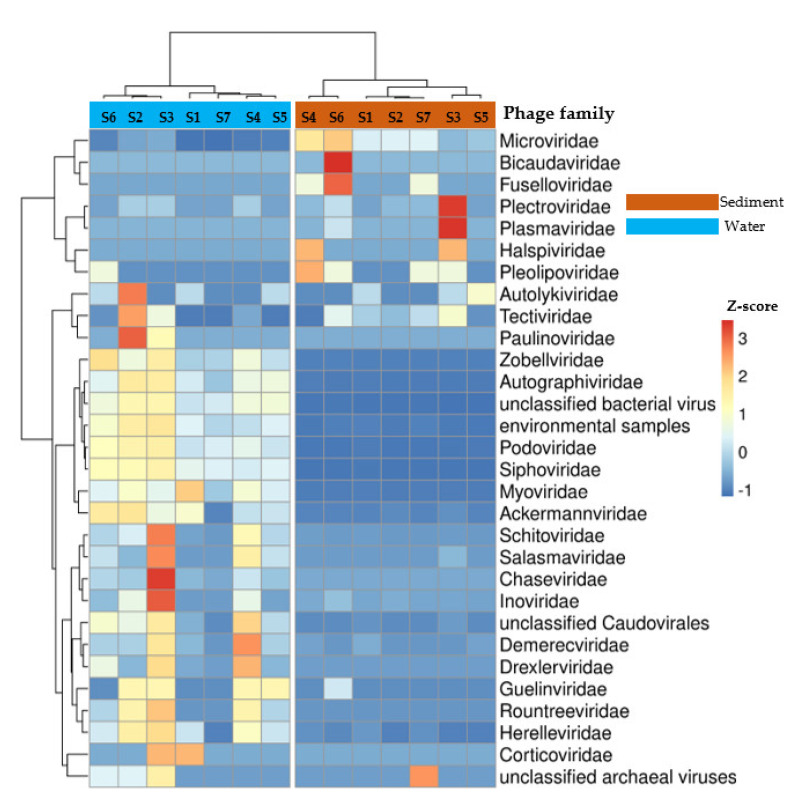
Hierarchical heatmap representing the relative abundance of the main phage families in the sediment and water samples of the seven stations (S1–S7). Number of contigs were normalized up to the smallest given contigs count for every samples. The relative abundance is represented by Z-score (based on the mean and standard-deviation (SD) of Z-scores for each family group in all samples). The stations and phage families were clus-tered using the Bray-Curtis distance, which is represented by a dendrogram on the top and right side of the graph. Z-scores are scaled by the relative abundance of phage families.

The hierarchical heatmap showed a distinctive taxonomic profile of phage families between sediment and water samples (Figure 2). Sequences assigned to *Microviridae*, *Tectiviridae*, *Pleolipoviridae*, and *Fuselloviridae* families were dominant in sediment samples, whereas sequences assigned as *Siphoviridae*, *Myoviridae*, *Podoviridae*, *Autographiviridae*, unclassified bacterial viruses, *Zobelliviridae* and *Ackermannviridae* were prevalent in water samples. Within the sediment and water samples, the taxonomic profiles of phages were relatively homogeneous (Figure 2).

### 3.3. Comparison of the Relative Abundances of Bacterial Communities (At Major Phyla Level) Determined by Phage-Host Prediction (HP) and 16S Metabarcoding (16S)

**Figure 3:**
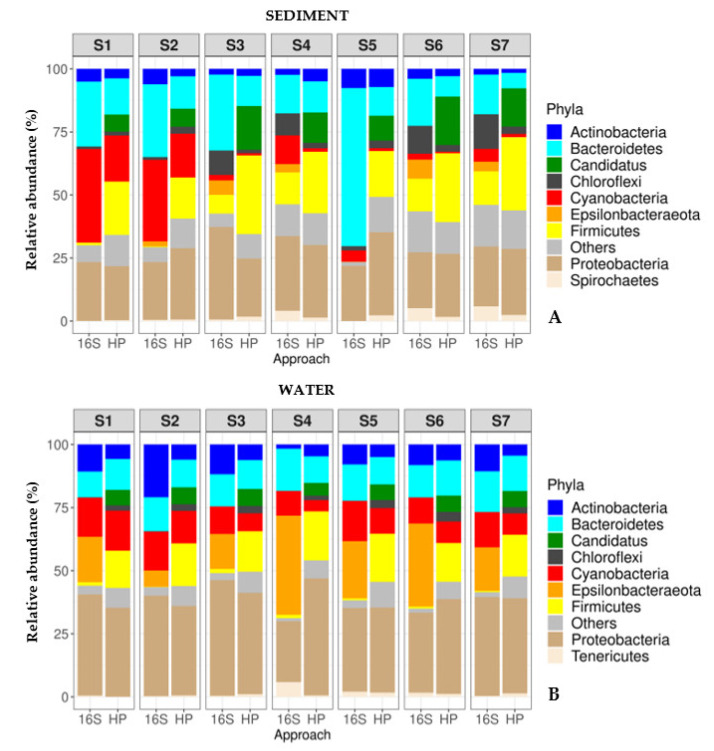
Stacked bar plot representing the relative abundances of bacterial communities (at phyla level) determined by 16S metabarcoding (16S) and phage-host prediction (HP) approach, in the sediment (**A**) and water (**B**) samples of the seven stations (S1–S7). Different bacterial phyla are represented by color code. “Others” in the plots represents a group of bacterial phyla with <1% relative abundances.

**Figure 4:**
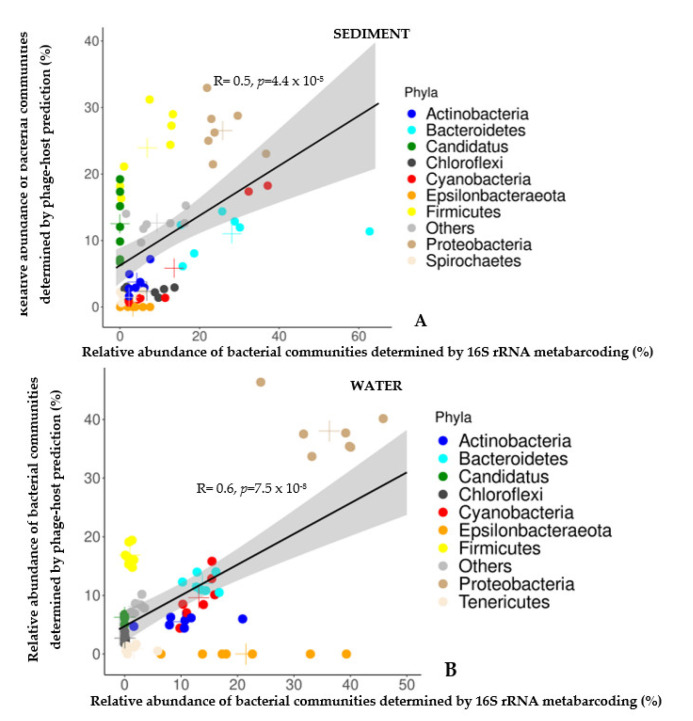
Scatter plot representing the correlation between the relative abundances of bacterial communities (at phyla level) determined by 16S rRNA metabarcoding (16S) and phage-host prediction (HP) approach, in the sediment (**A**) and water (**B**) samples of the seven stations (S1–S7). Each colored point corresponds to a single bacterial phylum. Colored crosses (**+**) indicate mean values of relative abundances. The grey areas represent a pointwise 95% confidence interval on the fitted values (regression line). “Others” in the figures represents a group of bacterial phyla with <1% relative abundances. R: Spearman rank correlation coefficients, *p* = *p* value.

In order to compare the relative abundance of bacterial communities obtained by host prediction (HP) and the 16S approach, a stacked bar-plot (Figure 3) and a scatter plot (Figure 4) were performed. These showed that for both sediment and water samples, the majority of bacterial phyla identified by HP were also detected by 16S (Figure 3A,B). 

The relative abundance of bacterial communities (at major phylum level) estimated by both approaches was significantly similar in sediment (Spearman correlation R = 0.5, *p-value* = 4.4 × 10^−5^) and in water samples (Spearman correlation R = 0.6, *p-value* = 7.5 × 10^−8^) (Figure 4A–B).

In sediment samples, bacterial communities identified by 16S were dominated by the phyla *Bacteroidetes* (28.2%), *Proteobacteria* (23.8%), *Cyanobacteria* (13.6%), *Firmicutes* (6.8%), and *Chloroflexi* (6.7%) (Figure 4A), while those identified by HP were dominated by the phyla *Proteobacteria* (26.5%), followed by *Firmicutes* (23.93%), *Canditatus* (12.5%), *Bacteroidetes* (11.1%), *Cyanobacteria* (5.8%) and *Actinobacteria* (3.7%) (Figure 4 A). In water samples, bacterial communities identified by 16S were dominated by the phyla *Proteobacteria* (36%), *Epsilonbacteraeota* (21.5%), *Bacteroidetes* (13.8%), *Cyanobacteria* (13.1%) and *Actinobacteria* (10.25%) (Figure 4B), whereas those identified by HP were dominated by the phyla *Proteobacteria* (38%), *Firmicutes* (16.9%), *Bacteroidetes* (12%), *Cyanobacteria* (9.6%), *Canditatus* (6.24%) and *Actinobacteria* (5.5%) (Figure 4B). 

Distinct variations in bacterial community composition at the phyla level were detected between the two approaches. In sediment samples, *Bacteroidetes* (28.2% vs. 11.1%) and *Epsilonbacteraeota* (3.21% vs. 0%) were more prevalent in bacterial communities identified by 16S, whereas *Firmicutes* (23.93% vs. 6.8%) and *Canditatus* (12.5% vs. 0%) were dominant in bacterial communities identified by HP (Figure 4A). In water samples, *Actinobacteria* (10.25% vs. 5.5%) and *Epsilonbacteraeota* (21.5% vs. 0%) were widespread in bacterial communities identified by 16S versus HP, whereas *Firmicutes* (16.9% vs. 0.9%), *Canditatus* (6.24% vs. 0%), and *Chloroflexi* (2.71% vs. 0.05%) were dominant in bacterial communities identified by HP versus 16S (Figure 4B).

## 4. Discussion

This study is the first to our knowledge to focus on the phage-host relationships of lagoon viromes. Investigating a viromic dataset with two metagenomic approaches, it resulted in a comprehensive map of phage identification that revealed *Siphoviridae*, *Myoviridae*, *Podoviridae* and *Microviridae* as the most dominant phage families, and their main putative bacterial hosts the phyla *Proteobacteria*, *Firmicutes* and *Bacteroidetes*. Using both methods (HP or 16S), distinct bacterial communities were found in water and sediment samples, and the same trend was found for phage communities. Of key interest, a taxonomic group of phages could be associated with a specific group of bacterial hosts. In sediment samples, a phage belonging to the *Microviridae* family was associated with *Bacteroidetes* and *Firmicutes* hosts, while in water samples, phages belonging to the order *Caudovirales* (families *Siphoviridae*, *Myoviridae* and *Podoviridae*) were associated with *Actinobacteria*, *Firmicutes* and *Proteobacteria* hosts. These findings suggest specific viral–bacterial community profiles depending on habitat type [34] and a close association between phages and their bacterial hosts. As phages are dependent on their hosts, their frequency and distribution are likely linked to that of their host [3]. They have also evolved with their host and often exhibit similar oligonucleotide frequency patterns with host genomes [3,10,11]. The “predicted” relative abundance of bacterial communities (at phylum level) showed a positive and significant correlation with the “real” relative abundance obtained in 16S metabarcoding, demonstrating the reliability of the predictive approach based on viral datasets generated by shotgun sequencing.

A challenge of the approach is that host prediction based on the genomic signature could not distinguish which phage infects which bacteria at species level, so we characterized the bacterial hosts of phages at the phylum level to avoid potential misclassification. In addition to this, studies focusing on the theoretical prediction of phage hosts implicitly assume that an individual phage infects a single host [8]. Yet in our study the majority of phage communities were grouped into four families represented by *Myoviridae*, *Podoviridae*, *Siphoviridae* and *Microviridae*, of which some taxa can infect a wide range of unrelated bacteria [35]. This may therefore bias the accuracy of bacterial host prediction. Another issue is that some phages can potentially be missed during virus-particle filtration, resulting in a biased representation of phage abundance and their associated hosts [7,36]. A final challenge is that the ability to classify phage sequences, whether to identify the taxa present or the putative functionality of a coding region, depends on the availability of representative viral sequences in the data repository used [37]. It is essential to consider database dependency and the limited number of characterized viral species when analyzing viromic datasets. 

Notably, there were distinct variations in the bacterial community composition identified by the two approaches at phylum level. For example, the phylum *Epsilonbacteraeota* was found exclusively in the bacterial communities identified by 16S, and *Canditatus* was found exclusively in bacterial communities identified by HP. A handful of previous studies [34,37,38] have compared predicted phage hosts based on viromes versus bacterial taxonomic profiles obtained by 16S, and have also demonstrated that the relative abundance of bacterial communities from the two approaches is not always similar. The nature and limitations of the approaches used (viral metagenomics/computational approach versus 16S metabarcoding) may explain some of the observed differences. A variety of factors may contribute to the discrepancies between these approaches, including limited availability of host genomes, misannotated or incomplete annotation of bacterial genomes used for host prediction (this was the case in our study), primer specificity/sensitivity, and taxonomical annotation bias due to uneven representation of bacterial genes in databases for 16S-based methods [39,40]. The phyla absent in bacterial communities obtained by 16S but present in those predicted by HP may have 16S rRNA gene sequences that do not perfectly match the primers used during the amplification step. Although 16S rRNA PCR primers are commonly referred to as “universal”, there is considerable sequence diversity in the 16S rRNA gene, even in the most well-conserved regions and among bacteria of the same species [41,42]. Despite these potential limitations, by combining a viral metagenomics-based approach with a computational tool, this study was able to provide a particularly thorough exploitation of the viromic dataset, allowing the first phage-host prediction in a lagoon ecosystem.

## 5. Conclusions

The immense diversity of viruses, especially bacteriophages, in different aquatic ecosystems is only beginning to be explored. Using a pioneering predictive approach combining a computational method and dedicated phage bioinformatics tools, this study shows that it is possible, to some extent, to improve our ability to identify a phage host without the need to culture each pair, a development that should contribute to a better understanding of viral ecology. Using a single viromic dataset, we were able to characterize phage communities and their putative hosts. The results found that phage-host prediction is reliable and allows the rapid identification of viral hosts, based on a comparison of the taxonomic profiles of the bacterial hosts obtained by the conventional metabarcoding approach targeting the 16S rRNA gene. To further improve its performance, it would be of interest to improve the annotation of representative bacterial genomes. The development of innovative bioinformatics methods that can be used in conjunction with high-throughput experimental approaches to predict phage-host dynamics promise to shed light on currently uncharacterized viromes in a variety of ecosystems.

## Data Availability

Not applicable.

## Supplementary Materials

The following supporting information can be downloaded at: https://www.mdpi.com/article/10.3390/v15010076/s1, Figure S1: Flow chart representing the overview of 16S metabarcoding and host prediction analyses; Figure S2: Principal coordinates plot (PCoA) representing the multivariate homogeneity of group dispersions (betadisper) in the water and sediment compartments; Figure S3: Bar plot showing the relative abundance (%) of the top 5 phage taxa at family level in the sediment and water samples of the seven stations (S1–S7); Table S1: Processing report of reads generated from 16S rRNA sequencing in the sediment and water samples of the 7 stations (S1–S7); Table S2: Number of contigs retrieved after metagenomic analysis with the corresponding phages contigs identified with VIRSORTER (category 1, 2 and 3) and VIBRANT.

## References

[1] Guerin E., Hill C. (2020). Shining Light on Human Gut Bacteriophages. Front. Cell. Infect. Microbiol..

[2] Suttle C.A. (2005). Viruses in the Sea. Nature.

[3] Clokie M.R., Millard A.D., Letarov A.V., Heaphy S. (2011). Phages in Nature. Bacteriophage.

[4] Breitbart M., Bonnain C., Malki K., Sawaya N.A. (2018). Phage Puppet Masters of the Marine Microbial Realm. Nat. Microbiol..

[5] Hobbs Z., Abedon S.T. (2016). Diversity of Phage Infection Types and Associated Terminology: The Problem with ‘Lytic or Lysogenic’. FEMS Microbiol. Lett..

[6] de Jonge P.A., Nobrega F.L., Brouns S.J.J., Dutilh B.E. (2019). Molecular and Evolutionary Determinants of Bacteriophage Host Range. Trends Microbiol..

[7] Moon K., Cho J.-C. (2021). Metaviromics Coupled with Phage-Host Identification to Open the Viral ‘Black Box’. J. Microbiol..

[8] Edwards R.A., McNair K., Faust K., Raes J., Dutilh B.E. (2016). Computational Approaches to Predict Bacteriophage–Host Relationships. FEMS Microbiol. Rev..

[9] Young F., Rogers S., Robertson D.L. (2020). Predicting Host Taxonomic Information from Viral Genomes: A Comparison of Feature Representations. PLoS Comput. Biol..

[10] Tan J., Fang Z., Wu S., Guo Q., Jiang X., Zhu H. (2022). HoPhage: An Ab Initio Tool for Identifying Hosts of Phage Fragments from Metaviromes. Bioinformatics.

[11] Lu C., Zhang Z., Cai Z., Zhu Z., Qiu Y., Wu A., Jiang T., Zheng H., Peng Y. (2021). Prokaryotic Virus Host Predictor: A Gaussian Model for Host Prediction of Prokaryotic Viruses in Metagenomics. BMC Biol..

[12] Ahlgren N.A., Ren J., Lu Y.Y., Fuhrman J.A., Sun F. (2017). Alignment-Free d^∗^_2_ Oligonucleotide Frequency Dissimilarity Measure Improves Prediction of Hosts from Metagenomically-Derived Viral Sequences. Nucleic Acids Res..

[13] Villarroel J., Kleinheinz K.A., Jurtz V.I., Zschach H., Lund O., Nielsen M., Larsen M.V. (2016). HostPhinder: A Phage Host Prediction Tool. Viruses.

[14] Galiez C., Siebert M., Enault F., Vincent J., Söding J. (2017). WIsH: Who Is the Host? Predicting Prokaryotic Hosts from Metagenomic Phage Contigs. Bioinformatics.

[15] Coutinho F.H., Zaragoza-Solas A., López-Pérez M., Barylski J., Zielezinski A., Dutilh B.E., Edwards R., Rodriguez-Valera F. (2021). RaFAH: Host Prediction for Viruses of Bacteria and Archaea Based on Protein Content. Patterns.

[16] Klindworth A., Pruesse E., Schweer T., Peplies J., Quast C., Horn M., Glöckner F.O. (2013). Evaluation of General 16S Ribosomal RNA Gene PCR Primers for Classical and Next-Generation Sequencing-Based Diversity Studies. Nucleic Acids Res..

[17] Callahan B.J., McMurdie P.J., Rosen M.J., Han A.W., Johnson A.J.A., Holmes S.P. (2016). DADA2: High Resolution Sample Inference from Illumina Amplicon Data. Nat. Methods.

[18] Quast C., Pruesse E., Yilmaz P., Gerken J., Schweer T., Yarza P., Peplies J., Glöckner F.O. (2013). The SILVA Ribosomal RNA Gene Database Project: Improved Data Processing and Web-Based Tools. Nucleic Acids Res..

[19] McMurdie P.J., Holmes S. (2013). Phyloseq: An R Package for Reproducible Interactive Analysis and Graphics of Microbiome Census Data. PLoS ONE.

[20] Bray J.R., Curtis J.T. (1957). An Ordination of the Upland Forest Communities of Southern Wisconsin. Ecol. Monogr..

[21] Bolger A.M., Lohse M., Usadel B. (2014). Trimmomatic: A Flexible Trimmer for Illumina Sequence Data. Bioinformatics.

[22] Chen S., Huang T., Zhou Y., Han Y., Xu M., Gu J. (2017). AfterQC: Automatic Filtering, Trimming, Error Removing and Quality Control for Fastq Data. BMC Bioinform..

[23] Andrews S. FastQC: A Quality Control Tool for High Throughput Sequence Data—ScienceOpen. https://www.scienceopen.com/document?vid=de674375-ab83-4595-afa9-4c8aa9e4e736.

[24] Bankevich A., Nurk S., Antipov D., Gurevich A.A., Dvorkin M., Kulikov A.S., Lesin V.M., Nikolenko S.I., Pham S., Prjibelski A.D. (2012). SPAdes: A New Genome Assembly Algorithm and Its Applications to Single-Cell Sequencing. J. Comput. Biol..

[25] Buchfink B., Xie C., Huson D.H. (2015). Fast and Sensitive Protein Alignment Using DIAMOND. Nat. Methods.

[26] Huson D.H., Auch A.F., Qi J., Schuster S.C. (2007). MEGAN Analysis of Metagenomic Data. Genome Res..

[27] Roux S., Enault F., Hurwitz B.L., Sullivan M.B. (2015). VirSorter: Mining Viral Signal from Microbial Genomic Data. PeerJ.

[28] Kieft K., Zhou Z., Anantharaman K. (2020). VIBRANT: Automated Recovery, Annotation and Curation of Microbial Viruses, and Evaluation of Viral Community Function from Genomic Sequences. Microbiome.

[29] Noguchi H., Park J., Takagi T. (2006). MetaGene: Prokaryotic Gene Finding from Environmental Genome Shotgun Sequences. Nucleic Acids Res..

[30] Eddy S.R. (2011). Accelerated Profile HMM Searches. PLoS Comput. Biol..

[31] Finn R.D., Bateman A., Clements J., Coggill P., Eberhardt R.Y., Eddy S.R., Heger A., Hetherington K., Holm L., Mistry J. (2014). Pfam: The Protein Families Database. Nucleic Acids Res..

[32] Jurtz V.I., Villarroel J., Lund O., Larsen M.V., Nielsen M. (2016). MetaPhinder—Identifying Bacteriophage Sequences in Metagenomic Data Sets. PLoS ONE.

[33] Benjamini Y., Hochberg Y. (1995). Controlling the False Discovery Rate: A Practical and Powerful Approach to Multiple Testing. J. R. Stat. Soc. Ser. B.

[34] Liu R., Qi R., Wang J., Zhang Y., Liu X., Rossetti S., Tandoi V., Yang M. (2017). Phage-Host Associations in a Full-Scale Activated Sludge Plant during Sludge Bulking. Appl. Microbiol. Biotechnol..

[35] Barylski J., Enault F., Dutilh B.E., Schuller M.B.P., Edwards R.A., Gillis A., Klumpp J., Knezevic P., Krupovic M., Kuhn J.H. (2020). Analysis of Spounaviruses as a Case Study for the Overdue Reclassification of Tailed Bacteriophages. Syst. Biol..

[36] Coutinho F.H., Gregoracci G.B., Walter J.M., Thompson C.C., Thompson F.L. (2018). Metagenomics Sheds Light on the Ecology of Marine Microbes and Their Viruses. Trends Microbiol..

[37] Bruder K., Maiki K., Cooper A., Sible E., Shapiro J.W., Watkins S.C., Putonti C. (2016). Freshwater Metaviromics and Bacteriophages: A Current Assessment of the State of the Art in Relation to Bioinformatic Challenges: Supplementary Issue: Bioinformatics Methods and Applications for Big Metagenomics Data. Evol. Bioinform..

[38] Ly M., Abeles S.R., Boehm T.K., Robles-Sikisaka R., Naidu M., Santiago-Rodriguez T., Pride D.T. (2014). Altered Oral Viral Ecology in Association with Periodontal Disease. mBio.

[39] Coclet C., Roux S. (2021). Global Overview and Major Challenges of Host Prediction Methods for Uncultivated Phages. Curr. Opin. Virol..

[40] Jo J.-H., Kennedy E.A., Kong H.H. (2016). Bacterial 16S Ribosomal RNA Gene Sequencing in Cutaneous Research. J. Investig. Derm..

[41] Peterson D., Bonham K.S., Rowland S., Pattanayak C.W., Klepac-Ceraj V., Deoni S.C.L., D’Sa V., Bruchhage M., Volpe A., RESONANCE Consortium (2021). Comparative Analysis of 16S RRNA Gene and Metagenome Sequencing in Pediatric Gut Microbiomes. Front. Microbiol..

[42] Větrovský T., Baldrian P. (2013). The Variability of the 16S RRNA Gene in Bacterial Genomes and Its Consequences for Bacterial Community Analyses. PLoS ONE.

