# Supplementary material for: Phage-Host Prediction Using a Computational Tool Coupled with 16S rRNA Gene Amplicon Sequencing
Source: Viruses. 2022 Dec 27;15(1):76. doi: 10.3390/v15010076 (PMC9862649; doi:10.3390/v15010076)
Supplement: Supplementary file 1 [file viruses-15-00076-s001.zip › Supplemetary Figures_Viruses.pdf]

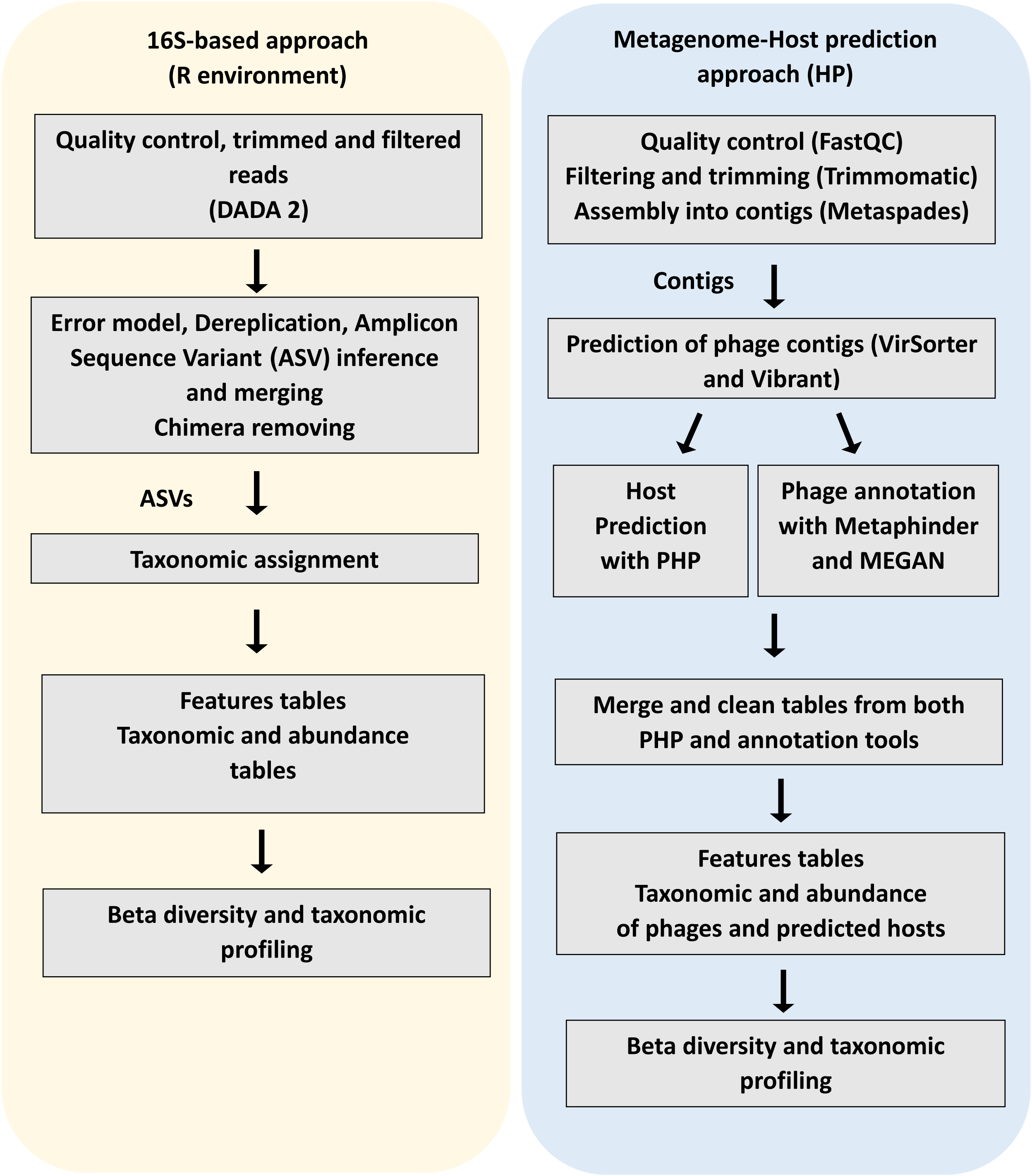

Figure S1. Flow chart representing the overview of 16S metabarcoding and host prediction analyses

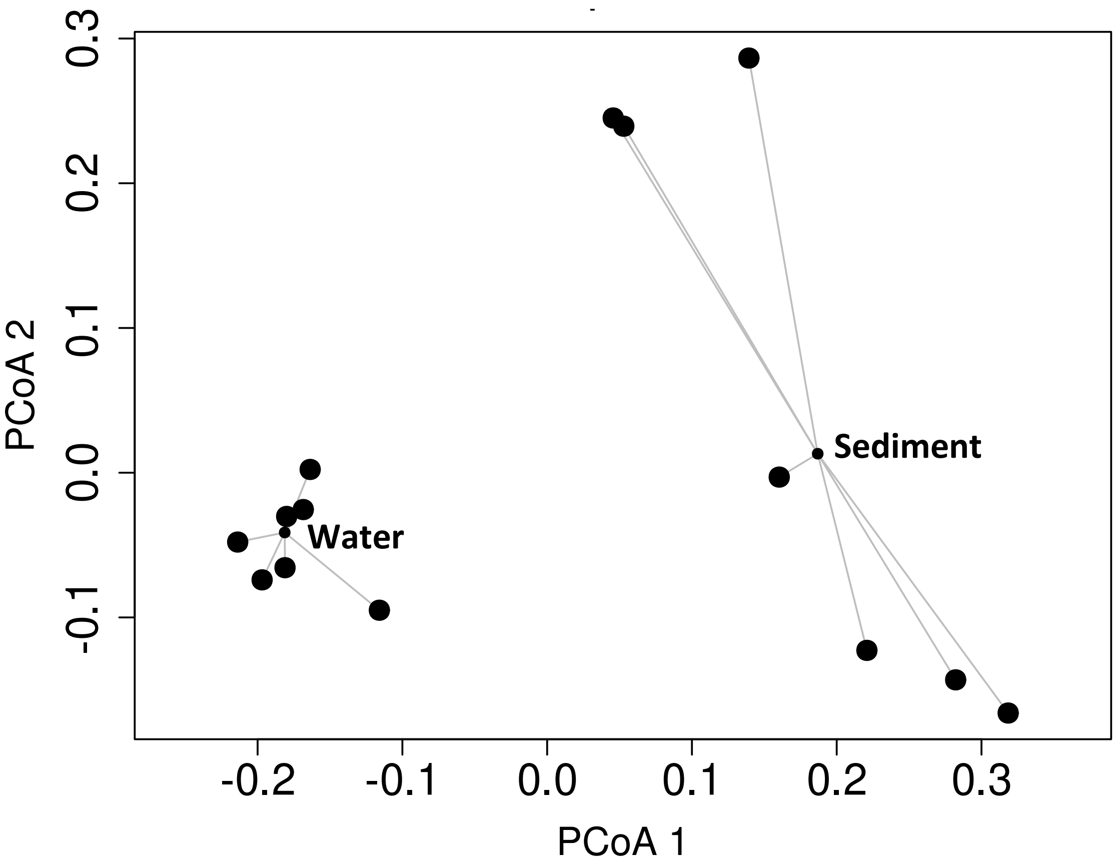

Figure S2. Principal coordinates plot (PCoA) representing the multivariate homogeneity of group dispersions (betadisper) in the water and sediment compartments. Each point represents a single station (seven per compartment). PCoA1 and PCoA2 are the first and second sort axes in the "betadisper" analysis, respectively.

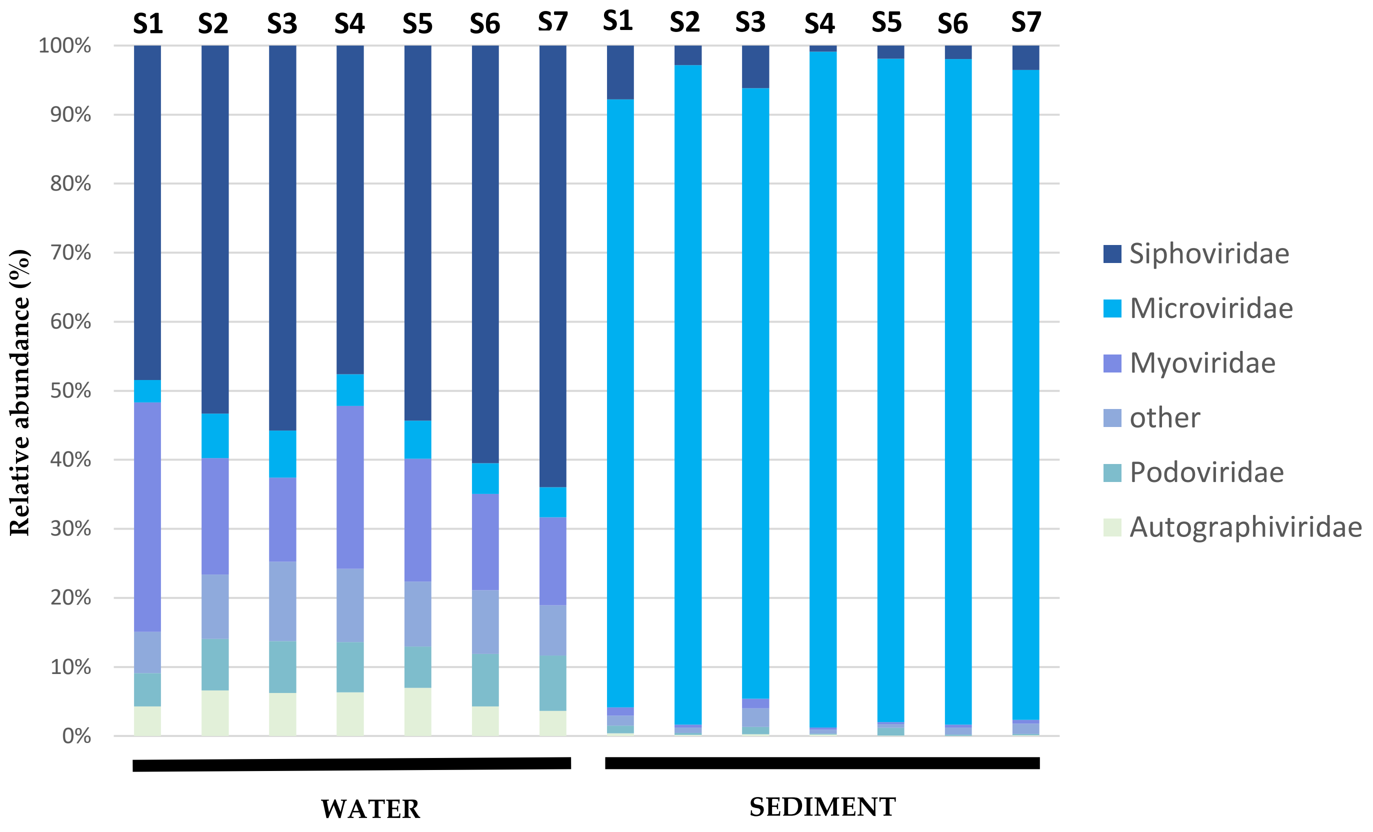

Figure S3. Bar plot showing the relative abundance (%) of the top 5 phage taxa at family level in the sediment and water samples of the seven stations (S1-S7). Viral families are represented by a color code.
