# Supplementary material for: Phage-Host Prediction Using a Computational Tool Coupled with 16S rRNA Gene Amplicon Sequencing
Source: Viruses. 2022 Dec 27;15(1):76. doi: 10.3390/v15010076 (PMC9862649; doi:10.3390/v15010076)
Supplement: Supplementary file 1 [file viruses-15-00076-s001.zip › Table S1.pdf]

Table S1. Processing report of reads generated from 16S rRNA sequencing in the sediment and water samples of the 7 stations (S1-S7). Each station is represented by triplicates.

Sample type	Stations	Input reads	Filtered	Denoised	Merged	Nonchimeric	Final_retained
SEDIMENT		92591	45953	44154	39870	38421	41.5
	**S1**	113798	56069	53690	47146	45599	40.1
		91475	45422	43217	38049	36399	39.8
		94271	46912	44406	38263	36659	38.8
	**S2**	78631	39643	37349	32302	31071	39.5
		48920	24610	22794	18513	17520	35.8
		82901	65842	62938	56002	53939	65.1
	**S3**	75430	59171	56067	48357	46311	61.4
		126307	98435	93261	78714	75252	59.6
		157408	123501	113300	90291	85943	54.6
	**S4**	83619	41616	37146	27964	26433	31.6
		59865	29268	25624	17842	16886	28.2
		44439	22375	20916	17967	17046	38.3
	**S5**	34598	17422	16207	13629	12992	37.5
		41391	21200	19695	16530	15520	37.5
		33066	16557	13727	9158	8655	26.2
	**S6**	66536	33497	29354	21017	20042	30.1
		79482	39178	35022	26166	25119	31.6
		76806	38913	34243	25108	23769	30.9
	**S7**	76945	38716	34093	24771	23448	30.5
		83179	42451	37240	28041	26412	31.7
WATER		165308	33418	31764	27522	26562	40.7
	**S1**	70413	37147	35642	31665	30531	43.3
		56959	29776	28853	26732	26366	46.3
		76449	37869	36476	33199	32524	42.5
	**S2**	72299	34413	33214	29886	29244	40.4
		91378	45607	44903	43011	41998	45.9
		102589	52060	50303	44881	43001	41.9
	**S3**	44044	21737	21404	20754	20483	46.5
		76657	38747	36959	32334	30890	40.3
		73246	38030	36732	32274	30064	41.1
	**S4**	35502	18527	17867	16019	15610	43.9
		109157	55046	53807	49180	47124	43.2
		1502003	50952	49029	44478	43479	42.6
	**S5**	113003	57484	56340	52023	48842	43.2
		96514	47651	45933	41774	40925	42.4
		75312	37122	35494	32383	31607	41.9
	**S6**	72989	36117	34487	31799	31184	42.7
		54316	27465	26158	23574	23004	42.3
		73237	36635	35432	32586	32040	43.7
	**S7**	82199	42207	40697	37412	36667	44.6
		78430	40639	39029	35811	35050	44.7
