# Supplementary material for: Phage-Host Prediction Using a Computational Tool Coupled with 16S rRNA Gene Amplicon Sequencing
Source: Viruses. 2022 Dec 27;15(1):76. doi: 10.3390/v15010076 (PMC9862649; doi:10.3390/v15010076)
Supplement: Supplementary file 1 [file viruses-15-00076-s001.zip › Table S2.pdf]

Table S2. Number of contigs retrieved after metagenomic analysis with the corresponding phages contigs identified with VIRSORTER (category 1,2 and 3) and VIBRANT in the sediment and water samples of the 7 stations (S1-S7)

Stations	Total contigs		Nb. of phage contigs identified with VIRSORTER		Nb. of phage contigs identified with VIBRANT	
	Sediment	Water	Sediment	Water	Sediment	Water
**S1**	27058	68116	507	1841	839	2352
**S2**	25471	147211	683	1717	896	2863
**S3**	89161	136906	589	1238	1262	2113
**S4**	61964	82840	489	1056	803	1710
**S5**	23164	173	346	714	518	1029
**S6**	125292	112680	616	1609	1252	2327
**S7**	90986	102767	401	1507	952	2111
**Total**	443096	650693	3631	9682	6522	14505
